# Dose-Escalated Robotic SBRT for Stage I–II Prostate Cancer

**DOI:** 10.3389/fonc.2015.00048

**Published:** 2015-04-07

**Authors:** Robert Meier

**Affiliations:** ^1^Swedish Radiosurgery Center, Seattle, WA, USA

**Keywords:** prostate cancer, stereotactic body radiotherapy, hypofractionation

## Abstract

Stereotactic body radiotherapy (SBRT) is the precise external delivery of very high-dose radiotherapy to targets in the body, with treatment completed in one to five fractions. SBRT should be an ideal approach for organ-confined prostate cancer because (I) dose-escalation should yield improved rates of cancer control; (II) the unique radiobiology of prostate cancer favors hypofractionation; and (III) the conformal nature of SBRT minimizes high-dose radiation delivery to immediately adjacent organs, potentially reducing complications. This approach is also more convenient for patients, and is cheaper than intensity-modulated radiotherapy (IMRT). Several external beam platforms are capable of delivering SBRT for early-stage prostate cancer, although most of the mature reported series have employed a robotic non-coplanar platform (i.e., CyberKnife). Several large studies report 5-year biochemical relapse rates which compare favorably to IMRT. Rates of late GU toxicity are similar to those seen with IMRT, and rates of late rectal toxicity may be less than with IMRT and low-dose rate brachytherapy. Patient-reported quality of life (QOL) outcomes appear similar to IMRT in the urinary domain. Bowel QOL may be less adversely affected by SBRT than with other radiation modalities. After 5 years of follow-up, SBRT delivered on a robotic platform is yielding outcomes at least as favorable as IMRT, and may be considered appropriate therapy for stage I–II prostate cancer.

## Background

Prostate cancer is the most common malignancy in men. An estimated 233,000 cases will be diagnosed in the United States in 2014 (1). PSA screening has led to earlier stage diagnoses; in 1998, 92% of prostate cancers were diagnosed with clinically organ-confined disease (2). Based on pre-treatment prognostic parameters, several systems have been proposed to stratify prostate cancer into differing risk groups; these are summarized in Table 1. In 2010, the seventh edition of the AJCC Staging Manual (3), added Gleason score and PSA to the TNM staging system, making these stage grouping roughly comparable to those of D’Amico and NCCN, with notable differences in the intermediate- and high-risk groups. NCCN also adds “very low-risk” and “very high-risk” categories.

**Table 1:** **Risk stratification systems**.

Clinical prognostic factors	D’Amico	NCCN	AJCC seventh edition
T1c, PSA < 10 ng/ml and PSA density < 0.15 ng/ml/g, Gleason ≤6 and ≤3 cores positive, and ≤50% cancer any core	Low-risk	Very low-risk	Stage I
T1–T2a, PSA < 10, Gleason ≤6	Low-risk	Low-risk	Stage I
T2b or PSA 10–20 or Gleason = 7 (single risk factor)	Intermediate-risk	Intermediate-risk	Stage IIa
T2b, PSA 10–20, Gleason = 7 (≥2 risk factors)	Intermediate-risk	Can shift to high-risk	Stage IIa
T2c and/or PSA > 20 and/or Gleason = 8–10	High-risk	High-risk	Stage IIb
T3b–T4, any PSA, any Gleason		Very high-risk	Stage III or IV

Nearly 50% of patients (4) diagnosed with prostate cancer fall in prognostic AJCC Stage I, which includes patients with a clinical stage of T1–T2a, PSA < 10, and Gleason 6. Active surveillance has become a suitable alternative for AJCC stage I, also referred to as “low-risk,” patients (5). The PIVOT trial randomized PSA-era diagnosed patients between radical prostatectomy and observation. The study was not designed to compare outcomes in the various risk groups, thus firm conclusions about subgroups cannot be made. Nevertheless, in the PIVOT trial, surgery was associated with 50 and 60% reductions in prostate cancer deaths for intermediate- and high-risk groups (D’Amico definition), respectively. This bolsters clinicians’ recommendations that these groups undergo definitive therapy.

According to the Prostate Cancer Clinical Guideline Panel of the American Urological Association in 2007 (6), treatment options that should be discussed include radical prostatectomy, radiotherapy with or without androgen deprivation, and active surveillance.

## Historical Evolution of Radiotherapy for Prostate Cancer

Radiotherapy was first used to treat prostate cancer in the first half of the twentieth century; the application of radium or kilovoltage therapy yielded disappointing results (7, 8). The development of megavoltage external beam platforms in the 1950s (9–11) allowed higher doses to be delivered, with encouraging outcomes. The next important development was CT imaging and computerized treatment planning, which facilitated three-dimension conformal external beam planning and intensity-modulated radiotherapy (IMRT). These more sophisticated treatment plans yielded better dose conformity to the target, allowing further dose-escalation. Conformal, dose-escalated techniques have yielded varying disease-free outcomes, approximately similar to those seen with radical prostatectomy (see Table 2), although not without toxicity.

**Table 2:** **bDFS outcomes for low-risk prostate cancer**.

Rx	Institution/author	Details	Pts	Median F/U years	5-Year bDFS and definition (%)			
					Nadir + 2	ASTRO	PSA ≥ 0.2	Average^a^
HDR + EBRT	Seattle, Kiel, Beaumont (12)	45–50 Gy + 2–4 fx boost	46	5		96		92
	CA endocurietherapy (13)	36 Gy + 5.5–6 Gy × 4 boost	70	7.25	93	90		
HDR alone	CA endocurietherapy (14)	6–7.25 Gy × 6	117^b^	8		96		97
	Beaumont (15)	9.5 Gy × 4	95^c^	4.2		98		
LDR	RTOG 9805 (16) phase II	145 Gy I-125 alone	95	5.3	99	93		88
	11 Inst meta-analysis (17)	I-125 and Pd-103 alone	1,444	5.25	86	88		
External beam	Clev Clin (18) hypofract	IMRT: 70 Gy, 2.5 Gy/fx	36	5.5	97	97		88
	MSKCC (19)	IMRT: 81 Gy, 1.8 Gy/fx	203	7	93	85	
	9 Instit meta-analysis (20)	3-D RT/IMRT: >72 Gy	70	5.7		79		
	9 Instit meta-analysis (20)	3-D RT/IMRT: 70–76 Gy	231	6.3	95			
	MDA rand dose-esc (21)	3-D conformal: 78 Gy	32	>5	93	92		
	MGH, Loma Linda (22)	Proton boost to 79.2 Gy	116	5.5		95		
Radic prost	Baylor: Hull (23)		299	3.9			92.5^d^	94
	Clev Clin, MSK: Kupelian (24)		524	5.5			92	
	Univ Penn: D’Amico (25)		322	5		88		
	Johns Hopkins: Han (26)		899	5.9			98	

*bDFS estimated based on proportions within each risk group*.

*^a^Weighted average of ASTRO bDFS or of stated bDFS definition in prostatectomy series*.

*^b^75% low-risk, 25% intermediate*.

*^c^Included T2b in low-risk group*.

*^d^PSA ≥ 0.4*.

*bDFS, biochemical disease-free survival; EBRT, external beam radiotherapy; IMRT, intensity-modulated radiotherapy; LDR, low-dose rate brachytherapy; HDR, High-dose rate brachytherapy*.

**Figure 1:**
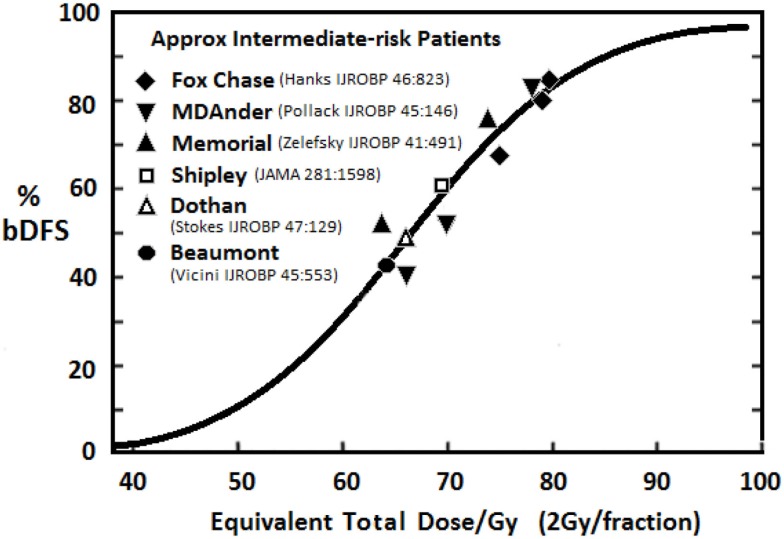
**Relationship between dose and 5-year freedom from PSA failure for intermediate-risk patients treated with EBRT [adapted from Fowler (29)]**.

Several randomized trials (22, 27, 28) have confirmed that dose-escalation yields improved relapse-free survival rates. Fowler’s dose–response analysis in intermediate-risk patients (29) (see Figure 1) indicates doses up to around 90 Gy are necessary to minimize recurrence rates. A meta-analysis of seven randomized dose-escalation trials yielded the same conclusion (30). A variety of strategies have been employed to further escalate dose and/or reduce toxicity to surround normal tissues.

Modern radiotherapy plans still had to account for variations in patient positioning, inaccuracies in treatment delivery, and internal organ motion. Radiation oncologists account for these uncertainties by adding a radial margin around the intended target, creating a “planning target volume (PTV).” This expanded target extends the high-dose treatment region into the surrounding normal structures. A PTV expansion of about 1 cm is required when skin marks are used for positioning. Set-up uncertainty can be reduced by placing gold fiducials in the prostate and imaging prior to treatment delivery. This does not account for movement within a given treatment session, or “intra-fractional” motion. Kupelian (31) demonstrated that in 15% of treatment sessions, the prostate moved more than 5 mm. A study from the Mayo Clinic (32) recommended a 5-mm margin to account for intra-fractional motion. The expanded PTV required in IMRT employing pre-treatment image guidance has limited the maximum safe dose around 82 Gy, if delivered at 2 Gy per fraction.

Proton therapy offers the prospect of prostate dose-escalation while limiting exposure to normal tissues. Proton beams deposit radiation until after passing beyond the target, where the dose then falls off rapidly. This reduces the radiation dose to normal tissues, potentially yielding fewer side effects. However, like IMRT, proton beam plans must account for prostate motion, thus the same large PTVs must be targeted. Also, since most proton beam plans employ only two beams, conformal dose sculpting around the prostate is not possible. While proton therapy reduces the volume of normal tissues receiving low dose radiation, large volumes of the rectum still receive high-dose radiation. In one study (33), protons yielded a 50% greater incidence of rectal toxicity compared to IMRT. The American College of Radiology Study (03–12) demonstrated (34) significant (8%) late grade 3+ rectal toxicity when proton dose was escalated to 82 Gy. Proton dose-escalation beyond 82 Gy is thus not possible with current technology, and long-term GI toxicity appears to be no better, and perhaps inferior to IMRT.

Transperineal ultrasound-guided brachytherapy allows the delivery of conformal, high-dose radiotherapy to the prostate, with a rapid dose fall-off outside of the implanted region. In low-dose rate (LDR) implants, 70–100 iodine-125 (I-125) or palladium-103 (Pd-103) sources are permanently placed within the prostate; these “seeds” slowly deliver radiation over the ensuing 2–6 months. For patients with low-risk prostate cancer, a single LDR implant (monotherapy) yields favorable long-term outcomes (35–37). Patients with intermediate- or high-risk disease usually require a 5-week course of external beam radiotherapy plus the LDR implant (38, 39). When post-implant dosimetry demonstrates that the prostate received a biologically equivalent dose (BED) of around 200 Gy, LDR brachytherapy yields exceptionally high relapse-free survival rates (40). This is equivalent to about 110 Gy at 2 Gy/fx, assuming α/β = 1.5. Unfortunately, toxicity following LDR brachytherapy appears to be greater than IMRT. Fox-Chase (41) reported 3-year grade 2+ GI and GU toxicities rates were three and fivefold greater following seed implants. Sanda’s patient-reported quality of life (QOL) study (42) did not directly compare treatments, however greater declines in urinary and bowel scores were observed following brachytherapy than after external beam radiotherapy.

## Hypofractionation

High-dose rate (HDR) brachytherapy has been used in the treatment of prostate cancer since the 1980s (43–50). Catheters are placed temporarily in the prostate, and then loaded with a high-dose Iridium-192 source, delivering a few fractions of very high-dose RT. Initial protocols employing HDR combined conventionally fractionated external beam RT with an HDR boost. More recent reports have employed HDR as monotherapy (14, 15, 45, 51–53). Adjusting for pre-treatment risk factors, these studies yield biochemical disease-free survival (bDFS) outcomes at least as favorable to those seen with LDR brachytherapy or conformal dose-escalated RT or IMRT (see Table 2). A prospective study from William Beaumont Hospital (15) comparing HDR monotherapy vs. LDR brachytherapy (Pd-103) showed a superior 5-year event-free survival (98 vs. 85%, *P* = 0.01) and a trend toward improved freedom from cancer failure (98 vs. 92%, *P* = 0.1) in the HDR cohort. The same group showed toxicity and QOL following HDR brachytherapy was more favorable than either LDR brachytherapy or conformal external beam RT (51, 54). These results suggest prostate cancer favorably responds to hypofractionated regimens.

Radiation oncologists fractionate RT dose to reduce toxicity to surrounding normal tissues. For most cancers, by delivering dose over several weeks, equivalent cancer-killing effect is achieved with reduced long-term toxicity. The effect of dose fractionation on both cancer and normal tissues can be estimated using the “linear-quadratic model.” In this model, the alpha–beta ratio reflects the response of normal tissues or cancers to changes in RT dose per fraction. Most cancers respond to RT as do rapidly dividing normal tissues (e.g., skin or mucous membranes), and thus have high α/β ratios, in the 8–12 Gy range (55). Tissues with lower α/β ratios are more sensitive to large dose per fraction (also known as hypofractionated) RT.

The results of HDR and other hypofractionated regimens led radiobiologists to reconsider α/β ratio of prostate carcinoma. Numerous studies have concluded that prostate cancer has an unusually low/ratio of about 1.5 Gy (29, 56–59). A pooled analysis (60) of 5,093 patients yielded a α/β ratio of 1.55 Gy. A low α/β ratio is consistent with other biologic properties of prostate cancer: an unusually long tumor doubling times (61), and a very low proportion of proliferating cells (62). If the α/β ratio for prostate cancer is smaller than the α/β ratios for late effects in the surrounding normal tissues (3–5 Gy), then a therapeutic gain could be achieved by hypofractionation. In this setting, larger doses per fraction should result in equivalent or improved cancer control with reduced toxicity (63–65).

Several prospective clinical trials have evaluated the efficacy of hypofractionated radiotherapy in organ-confined prostate cancer. A large prospective study from the Cleveland Clinic (66) demonstrated favorable relapse-free survival and low toxicity with 70 Gy given in 2.5 Gy fractions. A trial from Royal Adelaide Hospital in Australia (67) randomized 217 patients between 64 Gy in 2 Gy/fx vs. 55 Gy in 2.75 Gy/fx; these schedules are isoeffective if prostate α/β = 2.5. The hypofractionated arm showed a significantly better bDFS (53 vs. 43%), with equal toxicity in the two arms. In an Italian trial (68), 168 high-risk patients were randomized between 62 Gy in 3.1 Gy/fx vs. 80 Gy in 2 Gy/fx (isoeffective if prostate α/β = 1.8; both arms received 9 months of androgen ablation). Toxicities were equal. Overall relapse rates were equivalent, although improved cancer control was suggested if presenting PSA was 20 or less. Thus, the radiobiologic assertion that the α/β ratio for prostate cancer is low (1.5–1.8) has been confirmed by class 1 evidence.

Stereotactic body radiotherapy (SBRT) is the precise external delivery of very high-dose radiotherapy to targets in the body, with treatment completed in one to five fractions. Dose conformality is achieved using cross-firing ionizing radiation beams and image-guidance. By concentrating dose in the targeted cancer, SBRT maximizes cell-killing. Rapid dose fall-off minimizes radiation-related injury to adjacent normal tissues. Organ-confined prostate cancer should be ideally suited for SBRT as (I) dose-escalation should yield better outcomes; (II) the toxicity from treatment is due to high-dose radiation exposure to the organs immediately adjacent to the prostate; and (III) the unique radiobiology of prostate cancer favors hypofractionation.

## SBRT Platforms

Several external beam platforms can theoretically deliver stereotactic radiotherapy for prostate cancer. Table 3 summaries the capability of these devices. At a minimum, target localization prior to daily treatments is required. This can be performed using X-ray imaging of implanted fiducials, or on-board CT imaging. If intra-fractional image guidance is not employed, then at least 5 mm PTV expansions are required to account for target motion. If the target can be localized during treatment, then smaller PTV expansions can be employed, potentially reducing dose to surrounding organs. The accuracy of different real-time localization systems can vary considerably. For example, with the Novalis or Varian TrueBeam systems, typically localization and target positioning occurs only once prior to each treatment. With the Calypso system, the operator sets a threshold (typically 3–5 mm) beyond which treatment is interrupted and the patient positioning corrected. With the CyberKnife, continuous image acquisition and target correction occurs routinely; the Stanford group showed that when intra-fractional correction is done every 40 s, this device achieves sub-millimeter accuracy (69).

**Table 3:** **SBRT platforms**.

Platform	Description	Target localization method	Real-time correction	Rotational correction
CyberKnife	Linac on robotic arm, non-coplanar delivery, variable aperture or multileaf	Orthogonal X-rays, image implanted fiducials	Continuous, automated sub-millimeter correction	Yes, continuous automatic
Varian (Trilogy, TrueBeam etc.), w/Novalis, BrainLab	Linac on gantry. Multileaf collimator. Volumetric arc therapy available	Cone-beam CT, orthogonal X-rays, image implanted fiducials	Intermittent; tx interruption and manual correction	6-D couch available
Electa (Synergy, VersaHD etc.)	Linac on gantry. Multileaf collimator. Volumetric arc therapy available	Cone-beam CT	No	6-D couch available
Calypso	Used with gantry-based linacs	Implanted beacons provide real-time localization	Continuous; tx interruption and manual correction	No
Tomotherapy	Linac, helical delivery, multileaf collimator	Megavoltage CT	No	No

Correction for target motion must account for translational (i.e., anterior/posterior, right/left, and superior/inferior) motion. Since rotational motion, particularly pitch, can be substantial, correction for rotations may be beneficial, although this potential benefit has not been quantified. The use of multiple non-coplanar beams should yield better dose conformality than single-plane treatments. While non-coplanar delivery is possible for any platform, in practice, centers employing gantry-based linacs treat in a coplanar fashion, as non-coplanar delivery adds complexity and time. The intrinsically non-coplanar CyberKnife platform is reported (70) to yield more conformal treatment plans than IMRT.

## Clinical SBRT Outcomes

The first report (71) of hypofractionated stereotactic radiotherapy treated 40 low-risk patients using a conventional linear accelerator with daily localization of implanted fiducials. 33.5 Gy was delivered in five fractions to the prostate plus a 4–5-mm margin. Toxicities were acceptable. Four-year nadir + 2 bDFS was 90%, suggesting further dose-escalation would be beneficial.

The feasibility of SBRT employing further dose-escalation was first reported by King at Stanford University (72) using the CyberKnife platform. 36.25 Gy in five fractions of 7.25 Gy was delivered to the prostate plus a 3–5-mm margin. In the most recent update (73) of long-term outcomes in 67 patients, there were no grade 4+ toxicities. Two patients had a grade 3 urinary toxicity, and there were no grade 3 GI toxicities. Toxicities compared favorably to other radiation modalities. Five-year Kaplan–Meier PSA relapse-free survival was 94%. The majority of subsequent reports of prostate SBRT have employed the same platform. In a series of 304 patients treated with CyberKnife at Winthrop hospital, 5-year bDFS was 97, 90.7, and 74.1% in low-, intermediate-, and high-risk groups, respectively. Five grade 3 complications were reported, all GU, for an incidence rate of 2%. In a pooled analysis of eight institutions (74), 1,100 patients were treated with CyberKnife SBRT and followed a median of 36 months. Five-year bDFS rates were 95, 84, and 81% in low-, intermediate-, and high-risk groups, respectively. In a multi-center study (75), CyberKnife treated 129 intermediate-risk prostate cancers 40 Gy in five fractions of 8 Gy each, with only one grade 3 toxicity reported (GU: bladder injury). More recent reports (76, 77) have shown similar favorable outcomes with gantry-based platforms.

The mature series evaluating dose-escalated SBRT are summarized in Table 4. In low-risk patients treated to 35–36.25 Gy in five fractions, 5-year bDFS ranges from 94 to 97%. In Katz’s series (78) of 477 patients with a median follow-up of 6 years, 7-year actuarial relapse-free survival was 95.6%, confirming durable responses. In the low-risk patients treated in the eight-institution pooled analysis (74) and in Katz’ series (78), no difference in 5-year bDFS was seen when dose was escalated from 35 to 40 Gy. Sunnybrook (76) demonstrated 97% 5-year bDFS in 84 low-risk patients treated to 35 Gy in five fractions with a gantry-based system. In a series (77) of 98 low-risk patients treated to 40 Gy in five fractions with real-time tracking on a gantry-based linac, only one biochemical failure was reported at 5 years. Current data show no evidence of a dose–response beyond 7 Gy × 5 in low-risk patients. These SBRT outcomes compare favorably to the 92–97% 5-year bDFS typically reported with conventionally fractionated external beam radiotherapy (see Table 2).

**Table 4:** **Prostate SBRT series with mature follow-up**.

Institution	Platform	Dose fractionation	Median F/U years	Risk group	Pts	5-Year bDFS^a^ (%)
Virginia Mason (71)	Gantry-based linac	6.7 Gy × 5	3.4	Low	40	90^b^
Stanford (73)	CyberKnife	7.25 Gy × 5	2.7	Low and low–intermediate	67	94
Stanford, Naples (79)	CyberKnife	7–7.25 Gy × 5	5	Low and low–intermediate	41	93
Winthrop Hospital (78)	CyberKnife	7–7.25 Gy × 5	6	Low	324	97
				Intermediate	153	91
San Bortolo (80)	CyberKnife	7 Gy × 5	3	Low, intermediate, and high	100	94
Pooled eight institutions (74)	CyberKnife	36–40 Gy in 4–5 fxs	3	Low	641	95
				Intermediate	334	84
				High	125	81
Katz and Kang (81)	CyberKnife	7–7.25 Gy × 5	5	High	97	68
Multi-institution (82)	CyberKnife	8 Gy × 5	3	Intermediate	137	97
Sunnybrook (76)	Gantry-based linac	7 Gy × 5	4.75	Low	84	97
Twenty-first century (77)	Gantry-based linac	8 Gy × 5	5	Low	98	99

*^a^Nadir + 2 definitions*.

*^b^Four-year bDFS reported*.

*bDFS, biochemical disease-free survival; SBRT, stereotactic body radiotherapy*.

In intermediate-risk patients treated with SBRT, bDFS outcomes vary. In 153 stage IIa patients from Katz’s series (78), 7-year bDFS was 90%. In a multi-center study (82) of 137 intermediate-risk patients given 8 Gy × 5 fractions on the CyberKnife platform, 5-year bDFS was 96%. In a pooled analysis of eight institutions (74), 5-year bDFS in intermediate-risk patients was only 84%. However, those patients who received biologically higher doses (38 Gy in four fractions or 40 Gy in five fractions) had 5-year bDFS of 96.7%. The apparent improvement in bDFS in the higher-dose cohort was not statistically significant, and patient populations were not identical. Katz’s intermediate-risk group had 1/3 Gleason 4 + 3 and excluded patients with two high-risk features, while the multi-institutional study had 20% Gleason 4 + 3 patients, but included some patients with two high-risk features. Longer follow-up and comparisons of larger populations will be necessary to confirm trends suggesting dose-escalation beyond 7.25 Gy × 5 yields better relapse-free survival in intermediate-risk patients. These SBRT 5-year relapse-free survival rates compare favorably to fractionated EBRT (22, 83) outcomes, which are typically around 85%. The favorable 5–7-year bDFS rates seen following SBRT may prove clinically relevant, as IMRT bDFS rates for intermediate-risk patients steadily drop beyond 5 years. Even at dose levels of 86.4 Gy, 10-year relapse-free survival rate are around 75% (83).

Mature data evaluating SBRT in high-risk prostate cancer are limited. The largest series is a pooled analysis of eight institutions (74), in which 125 high-risk patients received CyberKnife with or without androgen deprivation therapy (ADT). Five-year bDFS was favorable at 81%. Katz (81) reported on a series of 97 high-risk patient treated with either five fractions CyberKnife (35–36.25 Gy) or CyberKnife boost (19–21 Gy in three fractions following 45 Gy pelvic RT). Forty-six of the 97 patients received ADT. Five-year bDFS was 68%. The addition of pelvic radiotherapy or ADT had no impact on relapse-free survival, although pelvic RT was associated with greater GI toxicity.

## Androgen Deprivation Therapy

Androgen deprivation therapy is routinely added to conventional RT in unfavorable intermediate-risk and high-risk prostate cancer patients (84). In intermediate-risk patients, RTOG 94-08 (85) demonstrated an overall survival benefit when 4 months of neoadjuvant hormone therapy (NHT) was added to 66.6 Gy of external beam RT. We now know higher external beam doses yields better outcomes. However, even with modern dose-escalated external beam RT, ADT appears to benefit unfavorable intermediate-risk patients (86). ADT reduces both micrometastatic and local disease burden; the latter effect may make up for radiotherapy doses that are inadequate to sterilize the primary disease site. In patients treated with brachytherapy (which delivers higher biologic doses to the prostate), a benefit from ADT is unclear, as there are studies (87, 88) showing conflicting results. The only study evaluating the impact of ADT on SBRT showed no benefit (81). Even short-term ADT is associated with hot flashes, erectile dysfunction, muscle loss, fat accumulation, increased cholesterol, and decreased insulin sensitivity (89, 90). If dose-escalation obviates the need for ADT in some subgroups, then SBRT may allow some patients to avoid the toxicity of ADT. More research is needed in this area.

## SBRT Toxicity

**Figure 2:**
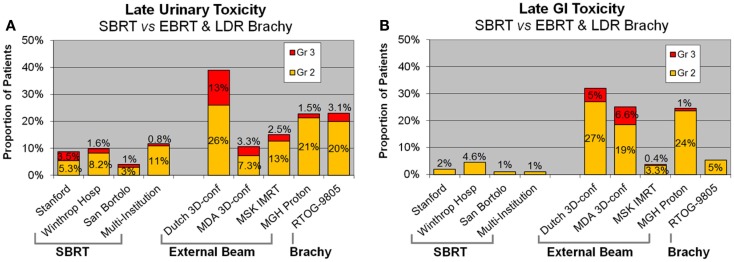
**Late urinary (A) and GI (B) toxicity rates following SBRT, external beam radiotherapy, and brachytherapy**. SBRT, stereotactic body radiotherapy.

Rates of late physician-reported GI and GU toxicities from mature SBRT series and from 3-D conformal, IMRT, proton, and LDR brachytherapy series are summarized in Table 5. Since median follow-up on the SBRT series is the 3–5-year range, these rates may underestimate the true rates of toxicities, as more toxicities may develop with longer follow-up. Nevertheless, Figure 2A, which illustrates the rates of grade 2+ toxicities for various modalities, suggests SBRT late urinary toxicity rates compare favorably to external beam. Late rectal toxicity rates appear to be consistently less than those seen with external beam radiotherapy (Figure 2B). These series employed a robotic non-coplanar delivery platform which corrected for target motion in real-time (CyberKnife), although recent reports of SBRT employing conventional gantry-based platforms (76, 77) also suggest favorable toxicity. A recent study (91) comparing Medicare claims found SBRT was associated with 38% more diagnoses of urethritis, incontinence, and obstruction, compared to IMRT. This study did not evaluate patients treated with G0339 and G0340 codes (used prior to 2014 with CyberKnife delivery), so the increased toxicity may be related to the differences in treatment technique and/or platforms. Finally, most SBRT series limited PTV doses to 35–40 Gy in five fractions. In a multi-center dose-escalation SBRT study (92), 5 of 91 patients treated to 50 Gy in five fractions required colostomy for rectal injury. This emphasizes the need to respect dose constraints for critical structures surrounding the prostate.

**Table 5:** **Toxicity rates for SBRT vs. EBRT, protons, brachytherapy**.

Technique	Institution	Details	Median F/U years	Pts	Late GU toxicity (%)		Late GI toxicity (%)	
					Gr2	Gr3	Gr2	Gr3
SBRT (CyberKnife)	Stanford (73)	7.25 Gy × 5	2.7	67	5.3	3.5	2.0	0
	Winthrop Hosp (78)	7–7.25 Gy × 5	5.0	304	8.2	1.6	4.6	0
	San Bortolo Hosp (80)	7 Gy × 5	3.0	100	3.0	1.0	1.0	0
	Multi-institutional (75)	8 Gy × 5	3.0	137	11.0	0.8	1.0	0
3-D-Conf RT	Dutch Random Trial (27)	78.0 Gy	4.2	333	26.0	13.0	27.0	5.0
	MDA Random Trial (93)	78.0 Gy	8.7	151	7.3	3.3	19.0	6.6
IMRT	Memorial SKCC (83)	86.4 Gy	4.4	478	13.0	2.5	3.3	0.4
Protons	MGH PROG (22)	79.2 Gy	8.9	196	21.0	1.5	24.0	1.0
LDR	RTOG 9805 (16)	145 Gy	8.1	94	20.0	3.1	5.0	0

*SBRT, stereotactic body radiotherapy; IMRT, intensity-modulated radiotherapy; LDR, low-dose rate brachytherapy; RT, radiation therapy*.

## Patient-Reported Toxicity

**Figure 3:**
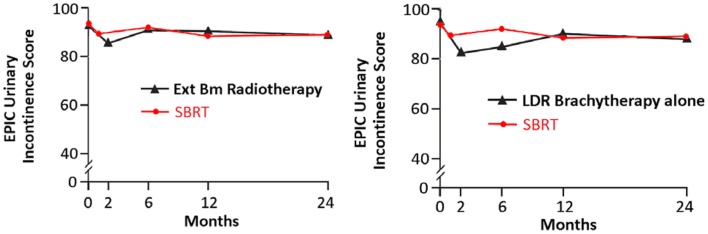
**EPIC urinary incontinence scores at baseline and at various intervals following treatment (months) from Sanda (96) (black: left graph is for external beam RT and right is for brachytherapy) and SBRT (red)**. SBRT, stereotactic body radiotherapy; RT, radiation therapy.

**Figure 4:**
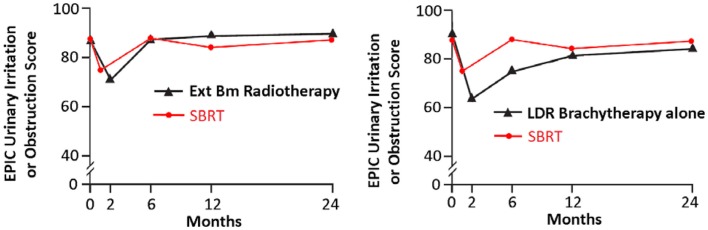
**EPIC urinary irritation/obstruction scores at baseline and at various intervals following treatment (months) from Sanda (96) (black: left graph is external beam RT and right is brachytherapy) and SBRT (red)**. SBRT, stereotactic body radiotherapy; RT, radiation therapy.

**Figure 5:**
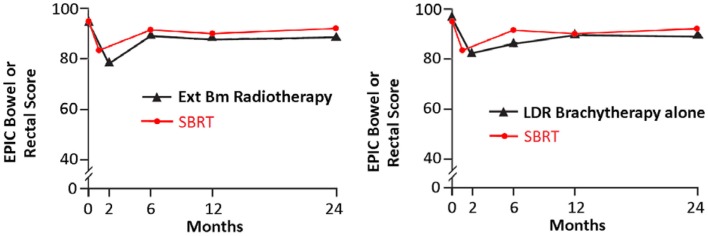
**EPIC bowel scores at baseline and at various intervals following treatment (months) from Sanda (96) (black: left graph is external beam RT and right is brachytherapy) and SBRT (red)**. SBRT, stereotactic body radiotherapy; RT, radiation therapy.

**Figure 6:**
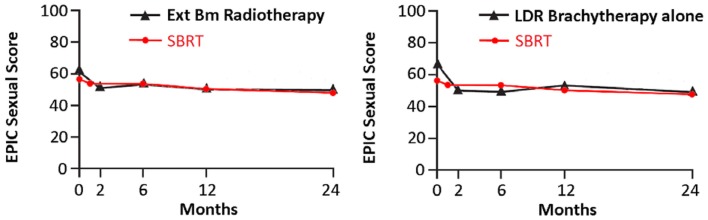
**EPIC sexual scores at baseline and at various intervals following treatment (months) from Sanda (96) (black: left graph is external beam RT and right is brachytherapy) and SBRT (red)**. SBRT, stereotactic body radiotherapy; RT, radiation therapy.

Following definitive therapy for prostate cancer, prospective patient-completed QOL questionnaires more accurately estimate treatment-related toxicity, compared to physician reports (94, 95). In Katz’ report of 304 patients treated with CyberKnife SBRT, urinary and bowel QOL decreased immediately following treatment, and then returned to baseline. Patient-reported QOL outcomes from a prospective multi-institutional study (82) of 309 patients treated with CyberKnife are illustrated in Figures 3–6 below. QOL outcomes of various organ domains from the validated EPIC instrument are superimposed on the benchmark external beam and brachytherapy outcomes reported in Sanda’s (96) study. Long-term changes in urinary incontinence scores following SBRT were similar to those observed in external beam and in brachytherapy (Figure 3). Urinary irritation/obstruction scores following SBRT appeared to be less adversely affected than after brachytherapy (Figure 4). While there were small changes in bowel QOL following SBRT (Figure 5), these declines appeared less prominent than following EBRT and brachytherapy. EPIC sexual score declined progressively during the 4 years after treatment (Figure 6). Because this methodology does not account for potential differences between SBRT and EBRT/LDR patient populations, no firm conclusions can be drawn. Nonetheless, these patient-reported SBRT QOL outcomes are encouraging.

## Cost Effectiveness

Although delivery of SBRT is technically more involved that IMRT, treatment is completed in only five fractions, rather than the 39–48 fractions required for IMRT. A Markov decision analysis model (97) showed the mean cost of $22,152 for SBRT vs. $35,431 for IMRT. Another study of Medicare claims (91) reported mean costs of $13,645 and $21,023 for SBRT and IMRT, respectively. These studies used SBRT billing codes, not the “robotic” G-codes used before 2014 for CyberKnife treatment. Since January 1, 2014, CyberKnife treatment also uses the same SBRT codes, thus these figures are relevant for robotic and non-robotic delivery. In addition to the cost for treatment, conventionally fractionated radiotherapy has a substantial time-cost to patients (98), which is mitigated by the far shorter treatment courses employed with SBRT.

## Ongoing Studies

The reported outcomes of SBRT in prostate cancer are derived from prospective non-randomized studies with the longest median follow-up extending to approximately 5 years. While these outcomes appear favorable relative to other radiation modalities, caution is warranted before concluding SBRT should supplant conventionally fractionated external beam RT. SBRT target doses, techniques of RT delivery, image-guidance approaches, and normal tissue constraints vary considerably between series, making comparisons difficult. While SBRT toxicities at 3–5 years appear favorable, higher rates of GU toxicity may be observed with longer follow-up. Finally, non-randomized comparisons are inherently uncertain. Firm conclusions about the efficacy and toxicity of SBRT relative to more conventional approaches await scrutiny by prospective randomized trials. Randomized trials registered on clinicaltrials.gov, ISRCTN registry, and cancerresearchUK.org are summarized in Table 6.

**Table 6:** **Randomized SBRT trials registered on clinicaltrials.gov, ISRCTN, and cancerresearchUK.org**.

Institution/study	Eligibility	Arms	Primary outcomes
Curie Institute Poland, NCT01839994	T1–T3a N0 M0	76–78 Gy, 2 Gy/fx	bDFS, toxicity
		50 Gy EBRT + 10 Gy × 2 SBRT/HDR boost	
University of Miami, NCT01794403	T1–T2 N0 M0, low-, intermediate-risk	70.2 Gy, 2.7 Gy/fx IMRT	2-year bDFS
		36.25 Gy, 5 fxs SBRT	
University Hosp Geneva, NCT01764646	T1–T3a N0 M0	36.25 Gy SBRT 9 days	Acute, late toxicity
		36.25 Gy SBRT once/week	
Swedish HYPO-RT-PC, ISRCTN45905321	Intermediate-risk	78 Gy, 2 Gy/fx RT	bDFS
		42.7 Gy, 6.1 Gy/fx	
Royal Marsden PACE, CRUKE/12/025	T1–T2 N0 M0	Prostatectomy vs. SBRT (36.25–38 Gy, 4–5 fxs)	5-year bDFS
		SBRT vs. conventional RT (78 Gy, 2 Gy/fx)	

## Conclusion

Stereotactic body radiotherapy offers a cost-effective alternative to external beam radiotherapy which is much more convenient for the patient. The radiobiology of prostate cancer would predict that this approach should yield superior outcomes compared to conventional protracted courses. For low-and intermediate-risk prostate cancer patients treated on a robotic, non-coplanar RT platform, 5-year relapse-free survival rates are at least equivalent, or possibly superior to conventionally fractionated RT. Physician-reported late urinary toxicity appears to be similar to external beam RT, and late GI toxicity appears to be less than with external beam and LDR brachytherapy. Patient-reported QOL outcomes show urinary and bowel function return to near baseline levels 2 years following robotic SBRT. Long-term changes in rectal QOL appear to be superior to those reported after IMRT or LDR brachytherapy. For high-risk prostate cancer, initial CyberKnife series suggest favorable outcomes. Emerging outcomes in low-risk patients treated on gantry-based platforms are similarly encouraging. A prospective randomized trial would be required to confirm these favorable SBRT outcomes relative to other modalities. But given these excellent cancer control rates and toxicity profiles, SBRT delivered on platforms which have real-time image guidance appears to be an acceptable approach for stage I–II prostate cancer. Further studies are also required to determine if similar favorable outcomes are possible with SBRT delivered on other platforms, and in patients with high-risk disease.

## Conflict of Interest Statement

The author declares that the research was conducted in the absence of any commercial or financial relationships that could be construed as a potential conflict of interest.
